# Bedtime routines, development, and caregiver educational attainment in toddlerhood

**DOI:** 10.3389/frsle.2023.1197297

**Published:** 2023-08-17

**Authors:** Joey Tsz Ying Lam, Ariel A. Williamson, Zainab Salih, Megan Heere, Jodi A. Mindell

**Affiliations:** ^1^Department of Psychology, Saint Joseph's University, Philadelphia, PA, United States; ^2^College of Education, Lehigh University, Bethlehem, PA, United States; ^3^Division of Pulmonary and Sleep Medicine, Children's Hospital of Philadelphia, Philadelphia, PA, United States; ^4^Department of Psychiatry, Perelman School of Medicine, University of Pennsylvania, Philadelphia, PA, United States; ^5^Department of Pediatrics, Lewis Katz School of Medicine, Temple University, Philadelphia, PA, United States

**Keywords:** bedtime routines, social-emotional development, behavioral intervention, education, toddlers

## Abstract

**Background:**

Implementing a consistent bedtime routine is an empirically supported intervention to improve sleep in toddlers, but little is known about its association with social-emotional outcomes, and among children living in lower socioeconomic status (SES) contexts.

**Objective:**

This study examined the longitudinal associations between bedtime routines, social-emotional development, and caregiver educational attainment in toddlers presenting to primary care.

**Method:**

Caregivers of 40 toddlers (*M*_age_ = 12.85 months, 57.5% female, 62.5% Black/African American) completed questionnaires on sociodemographic factors and child bedtime routine consistency at their 12-month well visit. At the 15- and 24-month well visits, data were collected on child bedtime routine consistency and social-emotional development, including the Brief Infant-Toddler Social and Emotional Assessment (BITSEA) for social-emotional problems and competency and the Ages and Stages Questionnaire (3rd edition; ASQ-3) to assess communication and personal-social skills.

**Results:**

Overall, the majority of families engaged in a consistent bedtime routine (≥5 nights/week) at all time points (63% at 12 months, 75% at 15 months, and 86% at 24 months). Controlling for concurrent bedtime routine consistency, toddlers with a more consistent bedtime routine at 12 months exhibited less dysregulation at age 15 months. Toddlers without a consistent bedtime routine at 15 months exhibited more externalizing and internalizing problems and dysregulation at 24 months. Furthermore, there was a significant interaction between bedtime routine consistency at 15 months and caregiver education for internalizing problems at 24 months, such that toddlers of caregivers with a high school education or less who lacked a consistent bedtime routine showed the most internalizing problems.

**Conclusion:**

Clinicians should consider recommending that families with toddlers incorporate a nightly bedtime routine not only to improve overall sleep health, but also to potentially optimize toddlers' positive social-emotional and behavioral trajectories, especially in families with lower educational attainment.

## Introduction

Sleep is essential for overall wellbeing and is especially important for optimal development in the first 5 years of life (Bathory and Tomopoulos, 2017). Healthy sleep in toddlers is associated with positive cognitive, language, and motor development, as well as emotional regulation (Maski and Kothare, 2013; Morales-Muñoz et al., 2020). A common, empirically supported behavioral intervention to improve sleep is the institution of a consistent bedtime routine, indicated as five or more nights per week (Mindell et al., 2015, 2018; Meltzer et al., 2021). In addition to sleep benefits, bedtime routines are hypothesized to foster parent-child attachment, health behaviors, prosocial development, and social-emotional development (Mindell and Williamson, 2018). However, few studies have investigated the potential benefits of bedtime routines for early social-emotional development (Kelly et al., 2013; Zajicek-Farber et al., 2014; Ren and Hu, 2019), as well as its relationship with socioeconomic factors. In particular, lower caregiver educational attainment has been linked to less consistent bedtime routine implementation in prior research (Hale et al., 2009; Williamson and Mindell, 2020), but has not been examined in relation to both bedtime routines and social-emotional development. As the first 3 years of life is a sensitive period for early childhood development (Black et al., 2017), understanding variation in bedtime routine consistency, caregiver educational attainment, and their relation to toddlers' social-emotional development can help guide efforts to promote early childhood wellbeing.

## Bedtime routines

General daily routines, including bedtime routines, are defined as a pattern of behaviors that are repeated consistently over time with active involvement of an adult (Fiese et al., 2002). From a behavioral perspective, a consistent routine acts as an environmental stimulus signaling the child to what is expected (Sytsma et al., 2001). Past research shows consistent routines are associated with decreased externalizing behaviors, as well as the development of better self-regulation and social skills (Bater and Jordan, 2017). Conversely, inconsistent routines or lack thereof make it difficult for toddlers to predict what activity comes next, which can yield unpredictable behavior such as tantrums, contributing to adverse child outcomes (Sytsma et al., 2001; Martin et al., 2012).

Based on the developmental theory that family routines positively impact child self-regulation (Sytsma et al., 2001; Martin et al., 2012), bedtime routines may be similarly beneficial. Bedtime routines are defined as a consistent set of caregiver-directed child activities, such as reading books, brushing teeth, and bathing, that occur every night in the hour before lights out (Mindell and Williamson, 2018). Furthermore, bedtime routine activities can be categorized into components, including nutrition, hygiene, communication, and physical contact, which can promote positive outcomes across developmental domains, including sleep, health, literacy, and attachment, among others. The benefits of implementing a consistent bedtime routine to child sleep are well-established in the literature. Past studies have found that implementing a consistent bedtime routine (i.e., five or more nights per week) is associated with an earlier bedtime, decreased sleep onset latency, decreased number and duration of night wakings, increased nighttime sleep duration, and fewer caregiver-reported sleep problems (Mindell et al., 2015, 2017, 2018; Fiese et al., 2021). A consistent bedtime routine also likely benefits young children's overall development. For example, institution of a language-based bedtime routine (e.g., parent-child book sharing, storytelling) at an early age is associated with better language ability (Hale et al., 2011), cognitive-academic skills (Câmara-Costa et al., 2021), and subsequent academic achievement (Câmara-Costa et al., 2021). Having a consistent bedtime routine is additionally associated with better executive functioning (i.e., inhibition-attention, working memory, and cognitive flexibility), school readiness, parenting style, and dental health (Kitsaras et al., 2018).

## Bedtime routines and social-emotional development

However, few studies have examined the potential benefits of bedtime routines on young children's social-emotional development. Social-emotional development refers to young children's ability to form and maintain meaningful relationships and express emotions. It encompasses prosocial behaviors (e.g., sharing and helping) and emotional and behavioral challenges (e.g., aggression and tantrums). One study of 2,977 children of low socioeconomic status (SES) backgrounds found that having a consistent bedtime routine at 36 months was associated with better concurrent emotional and behavioral regulation (Zajicek-Farber et al., 2014). Another longitudinal study found that irregular bedtimes were linked to child behavioral difficulties at ages 3, 5, and 7 (Kelly et al., 2013). Compared to those with consistent bedtimes, children with inconsistent and late bedtimes had higher scores on mother- and teacher-rated behavioral difficulties, such as hyperactivity, conduct problems, and peer problems. Moreover, children with irregular bedtime schedules at all three age points demonstrated a 3-fold increase in behavioral difficulties relative to those who only had one irregular bedtime schedule. In comparison, children who had an irregular schedule earlier in development followed by a consistent bedtime schedule by age 7 years old demonstrated improved emotional and behavioral outcomes. Although this study did not include bedtime routine implementation as a variable of interest, other studies have found that families who had irregular bedtime schedules were less likely to endorse consistent bedtime routines (Larsen and Jordan, 2020). Another study of school-aged Chinese children found that increased bedtime routine consistency was associated with better social skills, including communication, cooperation, and self-control, but not with problem behaviors (Ren and Hu, 2019). These studies all focused on preschool-aged and older children, with none involving toddlers.

## Current study

Overall, much of previous research has primarily focused on the positive impact of a consistent bedtime routine on sleep outcomes. Yet, little is known about the relationship between bedtime routines and social-emotional development, especially during toddlerhood, a critical period of development. Furthermore, few studies have examined bedtime routine consistency in relation to developmental outcomes. Given well-documented pediatric sleep health disparities (Williamson et al., 2019, 2021; Williamson and Mindell, 2020; El-Sheikh et al., 2022), understanding these associations while also examining socioeconomic factors, in this case lower caregiver educational attainment, that may impact family resources to implement child bedtime routines (Hale et al., 2009; Williamson and Mindell, 2020) is crucial to inform equitable interventions.

Thus, the overall goal of the current study was to examine the longitudinal associations of bedtime routine consistency and caregiver educational attainment with social-emotional development in toddlers. We examined associations among bedtime routine consistency at 12 and 15 months with toddler social-emotional development at 15 and 24 months, covarying for concurrent bedtime routine consistency. We hypothesized that implementing a more consistent bedtime routine would be associated with later social-emotional competencies, fewer social-emotional problems, as well as fewer communication and personal-social concerns longitudinally. We also explored associations between caregiver education and toddler social-emotional development, and whether the associations among bedtime routine implementation and social-emotional outcomes varied according to caregiver educational attainment.

## Method

### Participants

The current study includes control families participating in a larger randomized clinical trial examining sleep and developmental impacts of a bedtime routine intervention implemented in an outpatient primary care setting. The 50 control families were recruited at their toddler's 12-month well visit at one pediatric primary care office in Philadelphia, PA. Inclusion criteria were (1) primary caregiver or legal-guardian and (2) English-speaking. Of the 50 caregiver-child dyads, 37 (74%) completed the 15-month questionnaires: 6 (12%) did not return for their 15-month well visit and did not respond after multiple contact attempts, 5 (10%) moved away, and 2 (4%) declined to continue to participate. At the 24-month visit, 27 (54%) of the 50 caregiver-child dyads completed follow-up questionnaires: 13 (26%) did not return for their 24-month well visit and did not respond after multiple contact attempts, 5 (10%) moved away, and 5 (10%) declined to continue to participate.

**Figure 1:**
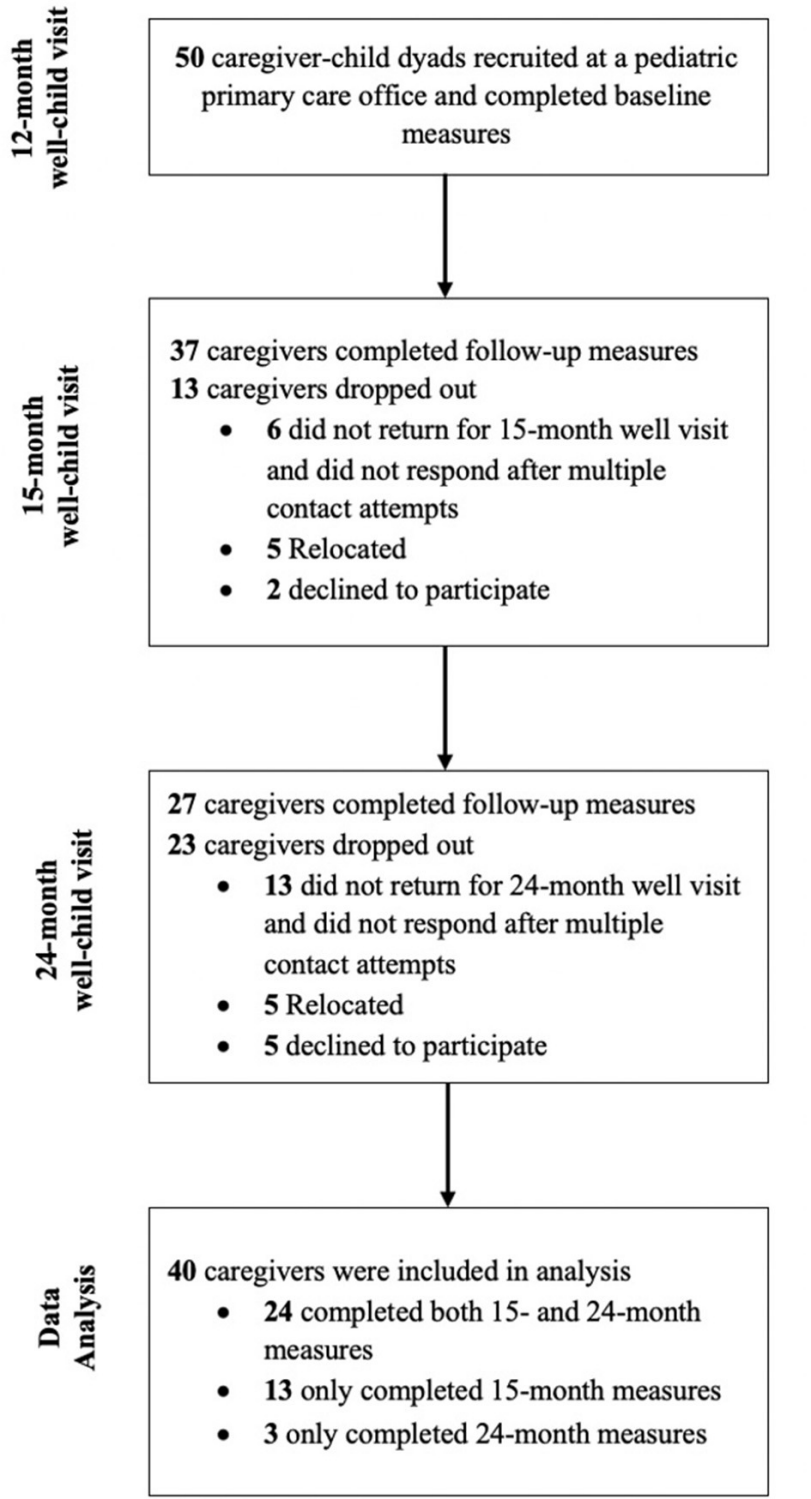
CONSORT flowchart of participants.

Inclusion in this study was based on completion of 15- and/or 24-month surveys. A total of 24 (60.0%) caregivers completed follow-up questionnaires at both their 15- and 24-month visits, 13 (32.5%) at their 15-month visit only, and 3 (7.5%) at their 24-month visit only. Thus, as shown in Figure 1, the final sample included 40 caregivers (95.0% parents; 80.0% female) of toddlers (M_*age*_ = 12.85 months; 57.5% girls). Overall, caregivers reported that 62.5% of toddlers were Black/African American, 12.5% White, and 27.5% Hispanic/Latinx (Table 1). Caregivers identified their race and ethnicity as 65.0% Black/African American, 10.0% White, and 27.5% Hispanic/Latinx.

**Table 1:** Sociodemographics (*N* = 40).

**Sociodemographic information**	**Mean (SD)**	**% (*n*)**
**Child age (months)**		
12-month visit (range 12.03–14.87)	12.85 (0.82)	
15-month visit (range 15.14–19.05)	16.69 (1.15)	
24-month visit (range 24.15–29.73)	26.16 (1.63)	
**Child sex**		
Boy		42.5 (17)
Girl		57.5 (23)
**Child race** ^*^		
Black/African American		62.5 (25)
White		12.5 (5)
Hispanic or Latinx		27.5 (11)
**WIC**		
Yes		67.5 (27)
No		32.5 (13)
**Caregiver age**		
18–24		25.0 (10)
25–29		35.0 (14)
30–39		35.0 (14)
40–49		2.5 (1)
50+		2.5 (1)
**Caregiver sex**		
Male		20.0 (8)
Female		80.0 (32)
**Caregiver race** ^*^		
Black/African American		65.0 (26)
White		10.0 (4)
Hispanic or Latinx		27.5 (11)
**Caregiver relation**		
Parent		95.0 (38)
Grandparent		2.5 (1)
Older sister		2.5 (1)
**Income**		
No income		22.5 (9)
$1–20,000		10.0 (4)
$20,001–35,000		10.0 (4)
$35,001–50,000		2.5 (1)
$50,001–75,000		5.0 (2)
$75,001–100,000		5.0 (2)
$100,001 or more		2.5 (1)
Prefer not to answer		42.5 (17)
**Caregiver education**		
High school/secondary school		50.0 (20)
Diploma/pre-university/junior college		7.5 (3)
Some college/university		25.0 (10)
College/university		15.0 (6)
Postgraduate		2.5 (1)
**Marital status**		
Single, never married		45.0 (18)
Single, previously married		2.5 (1)
Married		20.0 (8)
Unmarried, living with partner		32.5 (13)
**Occupation status**		
Employed full-time		40.0 (16)
Employed part-time		17.5 (7)
Homemaker/at-home parent/on maternity leave		22.5 (9)
Student		5.0 (2)
Unemployed/between jobs		15.0 (6)

^*^Race and ethnicity are included as socio-political constructs, not biological indicators.

### Procedure

This study was approved by a University Institutional Review Board. After obtaining informed consent, caregivers completed an online baseline questionnaire at their child's 12-month well visit, which consisted of sociodemographic questions and questions about their child's bedtime routine. When they returned for their child's 15-month and 24-month well visits, caregivers completed follow-up questionnaires regarding their child's bedtime routine and social-emotional development. Many of these questionnaires were completed as part of routine well visits. Caregivers were not compensated for their participation in this study.

#### Sociodemographic information and caregiver educational attainment

Sociodemographic information was collected at the 12-month well visit, including caregiver's age, relationship to child, marital and employment status, education level, household income, and Pennsylvania Special Supplemental Nutrition Program for Women, Infants, and Children (WIC) enrollment status. In addition, racial and ethnic backgrounds for the caregiver and child were obtained. In line with previous research (Hale et al., 2009; Williamson and Mindell, 2020), lower caregiver educational attainment was defined as having a high school education or less.

#### Bedtime routine consistency

At 12, 15, and 24 months, families reported on weekly bedtime routine consistency using the Brief Infant Sleep Questionnaire–Revised Short Form (BISQ-R SF; Mindell et al., 2019). The BISQ-R SF is adapted from the well-validated Brief Infant Sleep Questionnaire (BISQ; Sadeh, 2004), which has strong test-retest reliability and good correspondence with actigraphy-measured sleep and daily sleep logs. As in prior research, a consistent bedtime routine was defined as having a bedtime routine for five or more nights per week (Mindell and Williamson, 2018; Williamson and Mindell, 2020).

#### Social-emotional development

##### Brief Infant-Toddler Social and Emotional Assessment

To assess toddlers' social-emotional development, caregivers completed the 42-item Brief Infant-Toddler Social and Emotional Assessment (BITSEA; Briggs-Gowan, 2004) at their child's 15- and 24-month well visits. Caregivers rated their child behaviors for the past month on a 3-point Likert scale (0 = not true/rarely, 1 = somewhat true/sometimes, 2 = very true/often). The BISTEA is comprised of two scales: (1) the competence scale, consisting of 11 items measuring prosocial behaviors, empathy, and compliance, and (2) the problem scale, consisting of 31 items measuring six domains, including externalizing problems, internalizing problems, dysregulation, atypical behaviors (e.g., stereotypy, echolalia), maladaptive behaviors, and items of clinical significance (e.g., self-injury, pica). The BITSEA demonstrates excellent test-retest reliability (*r* = 0.82 to 0.92) and internal consistency (competence: α = 0.65; problem: α = 0.79; Briggs-Gowan, 2004).

##### Ages and Stages Questionnaire (3rd edition)

Caregivers completed the communication and personal-social subscales of the Ages and Stages Questionnaire (3rd edition; ASQ-3; Squires et al., 2001), an age-specific early childhood developmental screener that can identify potential developmental delays in communication, gross motor, fine motor, problem solving, and personal-social, at their child's 15- and 24-month well visits. Only the communication and personal-social subscales were administered as they reflect social-emotional constructs. The communication subscale (e.g., “When your child wants something, does she tell you by pointing to it?”) and the personal-social subscale (e.g., “Does your child play with a doll or stuffed animal by hugging it?”) each contain six items. Questions are scored with 10 points for “yes,” 5 points for “sometimes,” and 0 point for “not yet.” The ASQ-3 demonstrates strong test-retest reliability (*r* = 0.75 to 0.82) and adequate internal consistency (*r* = 0.51 to 0.87; Squires et al., 2001).

### Statistical analyses

Descriptive analyses (means and frequencies) were used to describe sociodemographic data, bedtime routine consistency at 12, 15, and 24 months, and social-emotional outcomes at 15 and 24 months. McNemar tests were conducted to examine the differences in bedtime routine consistency reported across the three time points. Chi-square analyses were conducted to examine the associations between caregiver education and bedtime routine consistency at each time point. Two-way ANCOVAs utilizing bedtime routine consistency at 12 and 15 months (0–4 nights/week vs. 5+ nights/week) and caregiver educational attainment (high school education level or less vs. some college or more) were conducted to examine the longitudinal associations with social-emotional competencies and problems (BITSEA) and communication and personal-social development (ASQ-3) at 15 and 24 months. In all models, bedtime routine consistency and caregiver educational attainment were entered simultaneously. We then entered bedtime routine consistency by caregiver educational attainment interaction terms to explore whether associations with social-emotional outcomes varied by caregiver education. Child sex was included in the analyses as a covariate. We also controlled for concurrent bedtime routine as a potential confound in examining the linkages between a bedtime routine at an earlier age and subsequent social-emotional functioning. Analyses were conducted using IBM SPSS version 28.0 with a significance level *p* < 0.05.

## Results

### Bedtime routines

At the 12-month visit, caregivers reported engaging their toddler in a bedtime routine on average 5.35 nights per week (*SD* = 1.99), with 63.2% (*n* = 24) consistently following a bedtime routine five or more nights per week. At the 15-month visit, caregivers reported a bedtime routine occurring on average 5.73 nights per week (*SD* = 1.56), with 75.7% (*n* = 28) indicating engaging in a consistent bedtime routine. At the 24-month visit, caregivers reported a bedtime routine occurring on average 5.85 nights per week (*SD* = 1.68), with 85.2% (*n* = 23) following a consistent bedtime routine on five or more nights per week. Further, McNemar tests showed no significant differences in caregivers following a consistent bedtime routine at 12 months and 15 months (*p* = 0.424), at 12 and 24 months (*p* = 0.375), and at 15 and 24 months (*p* = 0.070). Chi-square analyses revealed that toddlers whose caregivers had a high school education or less were less likely to implement a consistent bedtime routine at 15 months (58.8 vs. 90.0%), *p* = 0.028. However, caregiver educational attainment was not associated with bedtime routine consistency at 12 and 24 months.

### Social-emotional outcomes

At 15 months, the average score on the BITSEA competence scale was 17.78 (*SD* = 3.03). All toddlers had scores above the cutoff on the BITSEA competence scale, indicating that none demonstrated delays in social-emotional competencies. The average BITSEA problem scale score was 13.27 (*SD* = 8.10), with 29.7% (*n* = 11) receiving scores above the cutoff for possible social-emotional problems. Average scores were 35.28 (*SD* = 17.15) and 49.03 (*SD* = 12.81) on the ASQ-3 communication and personal-social subscales, respectively, with 18.9% (*n* = 7) and 8.1% (*n* = 3) receiving scores below the cutoff for communication and personal-social subscales, respectively, indicating potential developmental delays in these domains.

At 24 months, the average score on the BITSEA competence scale was 17.93 (*SD* = 4.19), with 11.1% (*n* = 3) receiving scores below the cutoff for potential delays in social-emotional competencies. Average BITSEA problem scale scores were 14.96 (*SD* = 9.80), with 33.3% (*n* = 9) receiving scores above the cutoff for possible social-emotional problems. Finally, average scores were 47.41 (*SD* = 19.38) and 45.93 (*SD* = 14.48) on the ASQ-3 communication and personal-social subscales, respectively, with 14.3% (*n* = 5) receiving scores below the cutoff on both subscales, indicating potential developmental delays in these domains (see Table 2).

**Table 2:** Social-emotional outcomes at 15 and 24 months.

**Social-emotional outcomes**	**15 months (*****n*** = **37)**		**24 months (*****n*** = **27)**	
	**Mean (SD)**	**Range**	**Mean (SD)**	**Range**
BITSEA competence	17.78 (3.03)	12–22	17.93 (4.19)	4–22
Above cutoff (%; *n*)	100.0% (*n* = 37)		88.9% (*n* = 24)	
Below cutoff (%; *n*)	0.0% (*n* = 0)		11.1% (*n* = 3)	
BITSEA problem	13.27 (8.10)	2–38	14.96 (9.80)	5–39
Externalizing	2.24 (2.68)	0–11	3.07 (3.11)	0–8
Internalizing	3.65 (2.97)	0–14	3.96 (3.23)	0–12
Dysregulation	3.41 (2.65)	0–8	3.81 (3.34)	0–12
Atypical	0.92 (1.50)	0–5	0.63 (1.08)	0–4
Maladaptive	0.51 (0.73)	0–2	0.48 (1.09)	0–4
Items of clinical significance	1.01 (1.21)	0–4	1.41 (1.28)	0–4
Above cutoff (%; *n*)	29.7% (*n* = 11)		33.3% (*n* = 9)	
Below cutoff (%; *n*)	70.3% (*n* = 26)		66.7% (*n* = 18)	
ASQ-3 communication	35.28 (17.15)	0–60	47.41 (19.38)	0–60
Above cutoff (%; *n*)	81.1% (*n* = 30)		62.9% (*n* = 22)	
Below cutoff (%; *n*)	18.9% (*n* = 7)		14.3% (*n* = 5)	
ASQ-3 personal-social	49.03 (12.81)	0–60	45.93 (14.48)	0–60
Above cutoff (%; *n*)	91.9% (*n* = 34)		62.9% (*n* = 22)	
Below cutoff (%; *n*)	8.1% (*n* = 3)		14.3% (*n* = 5)	

BITSEA, Brief Infant-Toddler Social-Emotional Assessment; ASQ-3, Ages and Stages Questionnaire (third edition).

### Bedtime routine consistency and toddler social-emotional outcomes

Controlling for child sex and concurrent (15-month) bedtime routine consistency, toddlers who had a consistent bedtime routine at 12 months had less behavioral dysregulation (*M* = 2.85) at age 15 months, compared to those with a bedtime routine less than five nights per week (*M* = 4.61), *p* =0.047. There were no differences in toddlers' social-emotional competence, externalizing problems, internalizing problems, maladaptive and atypical behaviors, communication, or personal-social concerns at age 15 months based on routine consistency at 12 months. Similarly, no significant associations were found between bedtime routine consistency at 12 months and all social-emotional outcomes at 24 months (see Table 3).

**Table 3:** ANCOVA results of social-emotional outcomes at 15 and 24 months by bedtime routine consistency at 12 and 15 months.

	**Bedtime routine consistency (12 months)**				**Bedtime routine consistency (15 months)**			
	**0–4 nights/week**	**5**+ **nights/week**	***F***	***p***	**0–4 nights/week**	**5**+ **nights/week**	***F***	***p***
	***M (SE)***	***M (SE)***			***M (SE)***	***M (SE)***		
15-month BITSEA competence	17.71 (0.89)	17.69 (0.66)	0.00	0.990				
15-month BITSEA externalizing	3.42 (0.75)	1.59 (0.55)	3.84	0.059				
15-month BITSEA internalizing	3.48 (0.80)	3.83 (0.59)	0.12	0.727				
15-month BITSEA dysregulation	**4.61 (0.68)**	**2.85 (0.50)**	**4.28** ^*^	**0.047**				
15-month BITSEA maladaptive	0.84 (0.20)	0.35 (0.15)	3.70	0.064				
15-month BITSEA atypical	0.84 (0.37)	0.93 (0.27)	0.04	0.852				
15-month ASQ-3 communication	40.31 (4.78)	34.81 (3.35)	0.88	0.356				
15-month ASQ-3 personal-social	52.33 (3.08)	49.58 (2.16)	0.52	0.473				
24-month BITSEA competence	20.12 (1.62)	16.78 (0.96)	3.00	0.101	15.52 (1.62)	18.84 (0.94)	3.02	0.099
24-month BITSEA externalizing	2.43 (1.32)	2.91 (0.78)	0.90	0.767	**5.30 (1.23)**	**2.01 (0.72)**	**5.10** ^*^	**0.037**
24-month BITSEA internalizing	4.36 (1.24)	4.35 (0.73)	0.00	0.990	**6.70 (0.86)**	**2.85 (0.50)**	**14.41** ^**^	**0.001**
24-month BITSEA dysregulation	3.88 (1.15)	4.20 (0.68)	0.05	0.820	**5.69 (0.98)**	**3.22 (0.57)**	**4.53** ^*^	**0.047**
24-month BITSEA maladaptive	0.08 (0.42)	0.75 (0.25)	1.80	0.197	1.10 (0.39)	0.30 (0.23)	2.95	0.103
24-month BITSEA atypical	0.87 (0.51)	0.58 (0.30)	0.23	0.637	0.81 (0.49)	0.47 (0.29)	0.35	0.562
24-month ASQ-3 communication	53.23 (7.98)	38.05 (4.70)	2.55	0.128	40.35 (8.18)	46.61 (4.76)	0.42	0.525
24-month ASQ-3 personal-social	52.55 (6.78)	39.84 (4.00)	2.47	0.133	41.02 (7.10)	45.43 (4.13)	0.28	0.605

ANCOVAs covary for child sex and concurrent bedtime routine consistency; caregiver educational attainment was also included in these models. BITSEA, Brief Infant-Toddler Social-Emotional Assessment; ASQ-3, Ages and Stages Questionnaire (third edition); M, means; SE, standard error.

^*^p < 0.05. ^**^p < 0.01. Bold indicates statistical significance.

Table 3 shows associations between bedtime routine consistency at 15 months and social-emotional outcomes at 24 months, controlling for child sex and concurrent (24-month) bedtime routine consistency. Toddlers with a consistent bedtime routine at 15 months exhibited fewer externalizing problems (*M* = 2.01 vs. 5.30; *p* =0.037), internalizing problems (*M* = 2.85 vs. 6.70; *p* =0.001), and less behavioral dysregulation (*M* = 3.22 vs. 5.69; *p* =0.047) at age 24 months. There was no significant variation in toddlers' social-emotional competence, maladaptive and atypical behaviors, communication, or personal-social concerns at 24 months, based on routine consistency at 15 months.

### Caregiver educational attainment and toddler social-emotional outcomes

Toddlers whose caregivers had a high school education or less exhibited more internalizing problems (*M* = 4.84 vs. 2.47; *p* = 0.030) and behavioral dysregulation (*M* = 4.83 vs. 2.63; *p* = 0.019) at age 15 months (see Table 4). Caregiver educational attainment was not associated with toddlers' social-emotional competence, externalizing problems, maladaptive and atypical behaviors, communication, or personal-social concerns at 15 months. Toddlers of caregivers with lower educational attainment exhibited significantly more internalizing problems (*M* = 5.95 vs. 2.76; *p* = 0.041), behavioral dysregulation (*M* = 6.33 vs. 1.75; *p* = 0.003), and maladaptive behaviors (*M* = 1.10 vs. 0.14, *p* = 0.034) at age 24 months. They also demonstrated lower communication skills (*M* = 34.46 vs. 51.46, *p* = 0.048). No significant associations emerged between caregiver educational attainment and other 24-month social-emotional outcomes (Table 4).

**Table 4:** ANCOVA results for social-emotional outcomes at 15 and 24 months by caregiver educational attainment.

	**Caregiver educational attainment**			
	**High school education or less**	**Some college or more**	***F***	***p***
	***M (SE)***	***M (SE)***		
15-month BITSEA competence	17.12 (0.12)	18.35 (0.71)	1.28	0.264
15-month BITSEA externalizing	2.74 (0.69)	1.83 (0.64)	0.87	0.357
15-month BITSEA internalizing	**4.83 (0.70)**	**2.65 (0.64)**	**4.98** ^*^	**0.032**
15-month BITSEA dysregulation	**4.62 (0.63)**	**2.38 (0.58)**	**6.37** ^*^	**0.017**
15-month BITSEA maladaptive	0.70 (0.19)	0.36 (0.17)	1.66	0.207
15-month BITSEA atypical	1.30 (0.34)	0.59 (0.31)	2.20	0.148
15-month ASQ-3 communication	31.86 (4.03)	40.70 (3.80)	2.39	0.132
15-month ASQ-3 personal-social	50.29 (2.55)	50.80 (2.41)	0.20	0.890
24-month BITSEA competence	16.54 (1.23)	18.90 (1.04)	2.09	0.164
24-month BITSEA externalizing	3.72 (0.97)	2.20 (0.81)	1.40	0.065
24-month BITSEA internalizing	**5.62 (0.89)**	**2.84 (0.75)**	**5.59** ^*^	**0.028**
24-month BITSEA dysregulation	**6.58 (0.82)**	**1.73 (0.69)**	**20.19** ^***^	**< 0.001**
24-month BITSEA maladaptive	**1.10 (0.32)**	**0.14 (0.27)**	**5.19** ^*^	**0.034**
24-month BITSEA atypical	0.84 (0.34)	0.47 (0.30)	0.61	0.445
24-month ASQ-3 communication	**34.46 (6.10)**	**51.46 (5.13)**	**2.13** ^*^	**0.048**
24-month ASQ-3 personal-social	36.28 (5.10)	49.80 (4.29)	4.00	0.059

ANCOVAs covary for child sex and concurrent bedtime routine consistency; bedtime routine consistency at prior timepoints was also included in these models. BITSEA, Brief Infant-Toddler Social-Emotional Assessment; ASQ-3, Ages and Stages Questionnaire (third edition); M, means; SE, standard error.

^*^p < 0.05. ^***^p < 0.001. Bold indicates statistical significance.

### Bedtime routine consistency, caregiver educational attainment, and social-emotional outcomes

As presented in Table 5, no significant interaction effects were found between bedtime routine consistency at 12 months and caregiver educational attainment across all social-emotional outcomes at 15 and 24 months.

**Table 5:** ANCOVA analyses for social-emotional outcomes at 15 and 24 months by bedtime routine consistency at 12 months and caregiver educational attainment.

	**Bedtime routine consistency (12 months)**					
	**0-4 nights/week**		**5**+ **nights/week**			
	***M (SE)***		***M (SE)***			
	**High school education or less**	**Some college or more**	**High school education or less**	**Some college or more**	***F***	***p***
15-month BITSEA competence	17.52 (1.22)	17.89 (1.31)	16.83 (1.02)	18.55 (0.88)	0.37	0.546
15-month BITSEA externalizing	4.28 (1.02)	2.56 (1.10)	1.69 (0.86)	1.49 (0.74)	0.67	0.421
15-month BITSEA internalizing	4.95 (1.09)	2.00 (1.18)	4.73 (0.91)	2.93 (0.79)	0.34	0.565
15-month BITSEA dysregulation	5.92 (0.93)	3.29 (1.01)	3.74 (0.78)	1.96 (0.67)	0.25	0.619
15-month BITSEA maladaptive	1.00 (0.28)	0.69 (0.30)	0.50 (0.23)	0.21 (0.20)	0.002	0.962
15-month BITSEA atypical	1.79 (0.51)	−0.11 (0.55)	0.95 (0.43)	0.90 (0.37)	4.06	0.053
15-month ASQ-3 communication	32.37 (6.22)	48.35 (7.29)	31.84 (5.20)	37.78 (4.50)	0.75	0.392
15-month ASQ-3 personal-social	52.23 (4.00)	53.43 (4.69)	49.01 (3.35)	50.16 (2.90)	0.02	0.900
24-month BITSEA competence	19.96 (2.81)	20.28 (1.56)	15.79 (1.33)	17.76 (1.36)	0.21	0.656
24-month BITSEA externalizing	1.93 (2.30)	2.94 (1.28)	4.12 (1.09)	1.69 (1.11)	1.35	0.261
24-month BITSEA internalizing	6.48 (2.15)	2.23 (1.20)	5.43 (1.02)	3.28 (1.04)	0.57	0.461
24-month BITSEA dysregulation	5.92 (2.00)	1.84 (1.11)	6.73 (0.95)	1.66 (0.97)	0.15	0.705
24-month BITSEA maladaptive	−0.02 (0.72)	0.18 (0.40)	1.35 (0.34)	0.15 (0.35)	2.23	0.152
24-month BITSEA atypical	1.16 (0.88)	0.58 (0.49)	0.77 (0.42)	0.38 (0.43)	0.03	0.869
24-month ASQ-3 communication	54.76 (9.98)	60.38 (7.67)	33.44 (6.14)	44.19 (6.31)	0.12	0.735
24-month ASQ-3 personal-social	51.76 (7.95)	52.62 (6.11)	32.55 (4.89)	47.16 (5.02)	1.34	0.260

ANCOVAs covary for child sex and concurrent bedtime routine consistency. BITSEA, Brief Infant-Toddler Social-Emotional Assessment; ASQ-3, Ages and Stages Questionnaire (third edition); M, means; SE, standard error.

**Figure 2:**
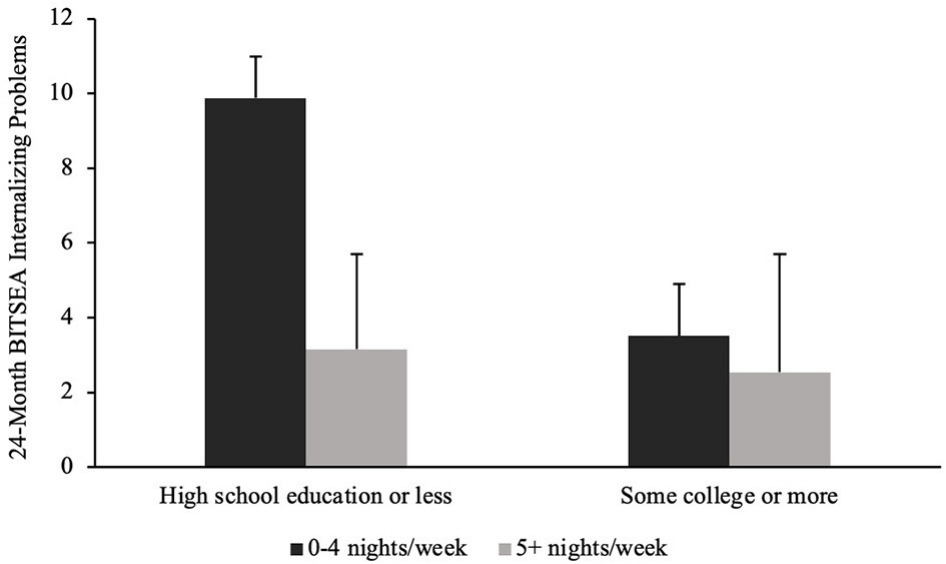
Twenty-four month internalizing problems by 15-month bedtime routine consistency and caregiver educational attainment.

There was a significant interaction between bedtime routine consistency at 15 months and caregiver educational attainment for toddlers' internalizing problems at 24 months, *F*_(1,18)_ = 7.41, *p* = 0.014 (see Figure 2). *Post-hoc* analyses revealed that toddlers whose caregivers had a high school education or less and lacked a consistent bedtime routine exhibited significantly more internalizing problems (*M* = 9.88) compared to those whose caregivers had a high school education or less but had a consistent bedtime routine (*M* = 3.52), *p* = 0.003. No significant interactions were found between bedtime routine consistency at 15 months and caregiver educational attainment for other 24-month social-emotional outcomes (see Table 6).

**Table 6:** ANCOVA analyses for social-emotional outcomes at 24 months by bedtime routine consistency at 15 months and caregiver educational attainment.

	**Bedtime routine consistency (15 months)**					
	**0-4 nights/week**		**5**+ **nights/week**			
	***M (SE)***		***M (SE)***			
	**High school education or less**	**Some college or more**	**High school education or less**	**Some college or more**	***F***	***p***
24-month BITSEA competence	13.26 (2.08)	17.76 (2.61)	18.44 (1.54)	19.23 (1.06)	0.88	0.361
24-month BITSEA externalizing	6.15 (1.59)	4.44 (1.99)	1.73 (0.81)	2.29 (1.17)	0.14	0.710
**24-month BITSEA internalizing**	**9.88 (1.11)**	**3.52 (1.39)**	**3.16 (0.82)**	**2.54 (0.57)**	**7.41**	**0.014** ^*^
24-month BITSEA dysregulation	9.55 (1.26)	1.83 (1.58)	4.87 (0.93)	1.58 (0.65)	3.37	0.083
24-month BITSEA maladaptive	2.15 (0.51)	0.06 (0.64)	0.49 (0.37)	0.11 (0.26)	3.13	0.094
24-month BITSEA atypical	1.57 (0.63)	0.05 (0.79)	0.42 (0.47)	0.51 (0.32)	1.79	0.198
24-month ASQ communication	20.38 (10.41)	60.78 (13.02)	43.05 (7.64)	49.89 (5.10)	2.89	0.105
24-month ASQ personal-social	27.36 (8.93)	55.03 (11.16)	41.73 (6.55)	49.24 (4.37)	1.42	0.248

ANCOVAs covary for child sex and concurrent bedtime routine consistency. BITSEA, Brief Infant-Toddler Social-Emotional Assessment; ASQ-3, Ages and Stages Questionnaire (third edition); M, means; SE, standard error.

^*^p < 0.05. Bold indicates statistical significance.

## Discussion

Overall, the majority of toddlers in this sample of families presenting to primary care had a consistent bedtime routine of five or more nights per week, between 63% at 12 months and 85% at 24 months. However, bedtime routine consistency was predictive of social-emotional outcomes. Toddlers with a consistent bedtime routine at 12 months of age had less behavioral dysregulation at 15 months. In addition, toddlers with a consistent bedtime routine at 15 months exhibited fewer externalizing and internalizing problems, as well as dysregulation, at 24 months, even after accounting for concurrent bedtime routine consistency. Bedtime routine consistency at 12 and 15 months was not associated with toddlers' communication and personal-social skills across time points. Lower caregiver educational attainment at 12 months of age was associated with later internalizing problems and dysregulation at 15 and 24 months, as well as maladaptive behaviors and lower communication skills at 24 months. Of note, there was a significant interaction between bedtime routine consistency and caregivers' education levels for future internalizing problems, such that toddlers whose caregivers had a high school education or less and lacked a consistent bedtime routine at 15 months displayed more internalizing problems at 24 months.

Our finding that toddlers with a consistent bedtime routine at 12 and 15 months exhibited less longitudinal behavioral dysregulation at 15 and 24 months and fewer externalizing and internalizing problems at 24 months aligns with previous findings in older children. For example, more consistent bedtime routines were linked with fewer externalizing behaviors and less bedtime resistance in a study of 120 predominantly White preschool-aged children (Larsen and Jordan, 2020). Another study of 87 predominantly Black/African American elementary school children from lower-SES backgrounds found that child-reported family routine (e.g., bedtime routine) moderated the relationship between teacher-reported child hyperactivity/impulsivity and oppositional defiant disorder symptoms (Lanza and Drabick, 2011). Additionally, in a population-based birth cohort study of 3,136 children in the Netherlands, Rijlaarsdam et al. (2016) also found that family regularity, including a consistent bedtime routine, at 2–4 years was longitudinally associated with a lower risk for child aggression in boys at age 6.

However, another study, using the same sample as Rijlaarsdam et al. (2016), found no longitudinal associations between family regularity at ages 2 and 4 and children's externalizing problems at age 6 (Hogye et al., 2022). It is possible that these studies yielded conflicting results because the latter study measured externalizing behaviors via child-report whereas other studies used caregiver-report to measure child externalizing problems. Collectively, however, most available studies suggest that a consistent bedtime routine is longitudinally associated with fewer social-emotional problems in older children. In our study, this relationship to outcomes at 2 years of age was stronger for bedtime routine consistency at 15 months versus 12 months.

Two underlying mechanisms may explain observed linkages between bedtime routine consistency and positive social-emotional development: (1) the regularity and predictive nature of a bedtime routine and (2) positive parent-child interactions during the routine. Presence of a consistent bedtime routine may act as an environmental stimulus to signal expectations to young children (Sytsma et al., 2001). In contrast, the absence of a bedtime routine or lack thereof, which may suggest a more chaotic household, could hinder toddlers' social-emotional development due to limited structure and predictability in daily activities (Bobbitt and Gershoff, 2016). Another possibility is that a bedtime routine generates rich opportunities for positive parent-child interactions, thereby fostering healthy social-emotional development (Mindell and Williamson, 2018). At bedtime, caregivers may have more opportunity to engage their child in interactive and stimulating activities, such as book-sharing and reading. Thus, caregiver involvement in a bedtime routine could include sensitive and responsive caregiving that cultivates secure child attachment (Bater and Jordan, 2017), thus facilitating healthy social-emotional development.

Contrary to our hypothesis, there were no longitudinal associations between bedtime routine consistency and toddlers' communication and personal-social skills across time points. These null results contrast with previous studies that found positive associations between bedtime routine consistency and these aspects of development. For example, a study of 228 school-aged Chinese children found that a consistent bedtime routine was associated with better social skills, including communication, cooperation, assertion, responsibility, empathy, engagement, and self-control (Ren and Hu, 2019). Other studies have found that consistent family routines, including a bedtime routine, are associated with better social skills in preschoolers (Koblinsky et al., 2006; Muñiz et al., 2014; Ferretti and Bub, 2017), whereas increased family chaos and less consistent routines are linked to decreased child prosocial behaviors (Bobbitt and Gershoff, 2016). Differences in child age could contribute to these discrepant findings, as these prior studies examined preschool-aged and older children, while our study focused on toddlers. It is also important to note that our small sample size might have yielded limited power to detect statistically significant associations with communication and personal-social skills. Furthermore, the ASQ-3 is a measure that focuses on developmental milestones and thus may not be as sensitive to more subtle differences in development, with modest accuracy in identifying early developmental delays (Sheldrick et al., 2020).

## Limitations

There are several limitations in the current study. First, the complete sample did not participate at all time points (70% at 15-months, 54% at 24 months). Although there were no significant sociodemographic differences between caregivers who completed the 15- and 24-month surveys and those who did not, as well as no differences in study participation rate of those with our without a consistent bedtime routine at baseline, our small sample size likely yielded limited power to detect statistical significance. This control sample was also drawn from a larger randomized trial, contributing to low power in this study. In addition, we relied on caregivers' report of their child's social-emotional development, which may be subject to bias and shared method variance. Future research may benefit from adopting a multi-informant approach (e.g., other family members and caretakers) and objective measures (e.g., behavioral observations) to assess both bedtime routines and social-emotional development.

The social-emotional measures were only administered at the 15- and 24-month visits, which limited our ability to control for toddlers' baseline social-emotional functioning and detect changes in functioning over time. Future research should administer social-emotional measures at baseline to better elucidate the difference in toddlers' social-emotional functioning at subsequent time points. Moreover, caregivers were not compensated for their participation. Although the primary care office administers many of these questionnaires as part of their routine visits, the lack of compensation may have contributed to the relatively high attrition rate. Also, generalizability of this study may be limited by the single primary care site from which families were recruited.

Additionally, although positive parent-child interactions during bedtime routines are postulated as an underlying mechanism to foster social-emotional development, we did not measure the quality of these interactions during the bedtime routine. Future research should examine these interactions to better understand their role in social-emotional development. It is also worth noting that institution of a consistent bedtime routine and social-emotional functioning may be bidirectionally linked (Williams et al., 2017; Quach et al., 2018). Thus, future studies should examine the reciprocal longitudinal interplay between bedtime routines and social-emotional development. Lastly, the current study did not measure caregivers' mental health (e.g., depression, anxiety) as a potential confound that might impede caregivers' ability to implement a consistent bedtime routine and might also negatively impact toddlers' social-emotional functioning. Future research should consider including caregivers' mental health as a contributor to these outcomes.

## Conclusion

This study is among the first to examine associations among bedtime routines, social-emotional development, and caregiver educational attainment in toddlers. Toddlers with a consistent bedtime routine early in development, at 12 and 15 months of age, exhibited fewer social-emotional problems longitudinally. We also found that toddlers whose caregivers had a high school education or less and lacked a consistent bedtime routine at 15 months showed significantly more 24-month internalizing problems, even after controlling for concurrent bedtime routine consistency. These findings highlight the promise of a consistent bedtime routine as an intervention to promote toddlers' social-emotional development, beyond established sleep benefits. Bedtime routine consistency may be especially beneficial for toddlers with caregivers of lower educational attainment, although this finding should be examined in future research with a larger sample.

Nonetheless, given the critical growth and development that occurs in the first few years of life (Black et al., 2017), clinicians should consider recommending caregivers incorporate a simple, adaptive bedtime routine during well-child visits. A bedtime routine is a feasible and cost-effective behavioral intervention that could help families optimize healthy social-emotional development in toddlers. Institution of a consistent bedtime routine may also buffer against the early onset of social-emotional problems, including among toddlers of caregivers with lower educational attainment.

## Data availability statement

The datasets presented in this article are not readily available because the datasets generated and/or analyzed during the current study are not available for use outside of Saint Joseph's University and Temple University at this time, due to the nature of the ethics board approvals and possible risk(s) to study participants as well as the confidentiality promised to them. Data may be made available from the corresponding author on reasonable request with permission of study investigators and ethics board approval. Requests to access the datasets should be directed to jmindell@sju.edu.

## Ethics statement

The studies involving human participants were reviewed and approved by Lewis Katz School of Medicine, Temple University, Philadelphia, PA, USA and Saint Joseph's University, Philadelphia, PA, USA. Written informed consent to participate in this study was provided by the participants' legal guardian/next of kin.

## Author contributions

JM, AW, and MH contributed to the conceptualization and design of the study. JL, ZS, and MH participated in data collection. JL and ZS organized the database. JL, JM, and AW contributed to the statistical analysis. JL wrote the first draft of the manuscript. JM and AW wrote sections of the manuscript and contributed to manuscript revision. All authors have read and approved the submitted version.
